# Groups clapping in unison undergo size-dependent error-induced frequency increase

**DOI:** 10.1038/s41598-017-18539-9

**Published:** 2018-01-16

**Authors:** Michael Thomson, Kennedy Murphy, Ryan Lukeman

**Affiliations:** Dept. of Mathematics, Statistics, and Computer Science, St. Francis Xavier University, P.O Box 5000, Antigonish, NS B2G 2W5 Canada

## Abstract

Humans clapping together in unison is a familiar and robust example of emergent synchrony. We find that in experiments, such groups (from two to a few hundred) always increase clapping frequency, and larger groups increase more quickly. Based on single-person experiments and modeling, an individual tendency to rush is ruled out as an explanation. Instead, an asymmetric sensitivity in aural interactions explains the frequency increase, whereby individuals correct more strongly to match neighbour claps that precede their own clap, than those that follow it. A simple conceptual coupled oscillator model based on this interaction recovers the main features observed in experiments, and shows that the collective frequency increase is driven by the small timing errors in individuals, and the resulting inter-individual interactions that occur to maintain unison.

## Introduction

The emergence of collective synchrony among interacting units underpins an array of natural phenomena^1^, such as the firing of pacemaker cells of the heart^2^, neural networks governing rhythmic human behaviours^3^, and fireflies flashing in unison^4^. The mathematical framework of coupled oscillators^5–8^ is typically used to study these processes, with the focus on the conditions and specific interactions that permit synchrony to emerge. Because many natural phenomena studied from this perspective have a stable period of oscillation, relatively little attention is focused on the behaviour of the system once synchrony is established.

One easily recognizable example of emergent synchrony is in groups of people clapping collectively. Under direction to clap in unison, such groups can quickly and reliably attain synchrony (synchronized with respect to both phase and frequency). However, in certain contexts, synchronous clapping can also emerge as a self-organized phenomenon. Neda *et al.* ^9^ showed that asynchronous applause following a performance transitioned temporarily to highly synchronous applause (a phenomenon occurring only in certain cultures). In this instance, synchrony was hypothesized to be dependent on the dispersion of natural clapping frequencies in the group, and the audience collectively attained synchrony via reduced dispersion by switching to an alternate low-frequency mode of clapping. An alternate explanation^10^ for the spontaneous emergence of order in audience applause used a decision-based queueing approach to suggest that synchrony emerges out of a sufficiently large desire for companionship behaviour relative to individualistic behaviour. Applause has also been studied from a social contagion perspective^11^, focusing on recruitment of applauding individuals in time, and not on synchronous behaviour.

In the case of synchronous applause, the synchrony is transient, being lost and regained as time progresses. Further, the loss of synchronization is caused by a reduction in period (i.e., increase in frequency), which causes an increase in dispersion of frequencies^9,12^. This was hypothesized to be due to a collective desire of the audience to increase the average noise intensity, possible primarily by speeding up the rhythm (more claps per unit time produces more total sound). In this study, we study the dynamics of synchronous clapping mechanistically: we seek to establish, through experiment and modeling, the inter-individual interactions governing synchronous clapping and observe what group-level features arise from these interactions, as opposed to assigning a hypothetical collective desire in the group that drives behaviour (beyond maintaining unison). Unlike other work, our primary focus lies in the collective features of the group after order is established, as opposed to the emergence of synchrony. In fact, the collective behaviour in the synchronous phase contains interesting dynamics, and provides information key to determining the inter-individual interactions driving the phenomenon.

At the individual level, humans (and a few other species) regularly engage in rhythmic behaviors and are able to readily entrain their behavior to match isochronic, or evenly paced stimuli, such as in rhythmic clapping, but also, e.g., in dancing and singing. The timing task of coordinating rhythmic perception and action, termed *sensorimotor synchronization* has been studied extensively, especially in tapping experiments, wherein participants respond to various rhythmic stimuli by tapping a key in time with their finger. Researchers have proposed a variety of models to explain the mechanism by which humans maintain synchronization through some form of error-correction (e.g., phase-correction^13^, period-correction, or both;^14^ see ref.^15^ for a review). Generally, individuals tend to a ‘copying effect’, tending to repeat the previous interval, although variability in response can cause instability. To counteract this instability, a ‘central tendency effect’ can be employed, whereby individuals average information over a number of previous periods. The two mechanisms are hypothesized to act in concert to explain synchrony in beat-based stimuli, along with phase and period correction mechanisms^16^.

When this individual rhythmic behaviour is embedded within the collective, additional complexity arises because other individuals are presumably providing some rhythmic stimuli. This creates a network of inter-individual interactions that can evolve in time, and leads to emergence of self-organization and collective dynamics not present at the individual level. Collective clapping is therefore a valuable example to study how humans can organize rhythmically. Because the mode of communication can be isolated to aural interactions (a discrete set of claps for each individual forms the totality of available information), the phenomenon is amenable to a mathematical modeling approach. Yet, aside from the aforementioned analyses of synchrony emergence in audiences and in (nonsynchronous) applause recruitment, there has been relatively little mathematical focus into how groups of humans can establish and maintain synchronous behaviour. From the sensorimotor synchronization perspective, studies have been extended from individuals to pairs^17^: participant pairs were asked to tap rhythmically with bidirectional coupling (hearing each others taps). Participants tended to mutually adjust their period on a tap-to-tap basis and entrain to one another, without any clear leader-follower relationship. In related work^18^, a weakly coupled oscillator model was built to study the observed tapping behaviour of the pairs. Using both period and phase coupling, 4 coupling constants were introduced and fit to a set of experiments, but the insight provided by this model is limited when scaling to larger groups, due to proliferation of parameters. The degree to which observations in single-and pair-participant tapping studies applies to a different task (clapping) in a different context (within a larger group) is unclear.

In this study, we record groups (from a few individuals to over two hundred) instructed to clap in unison. We find that, remarkably, every single group we analyzed (over thirty in total) tended to increase the frequency of the collective rhythm after synchrony is established. Furthermore, this increase occurs more rapidly as the group size increases. These observations, together with other metrics, are used to test two alternate hypotheses for the mechanism governing the collective behaviour. We ask whether the observed frequency increase is consistent with an innate, individual tendency to ‘rush’, versus arising as an epiphenomenon of the mode of interaction used to maintain collective synchrony in the face of small individual errors in timing.

## Results

**Figure 1:**
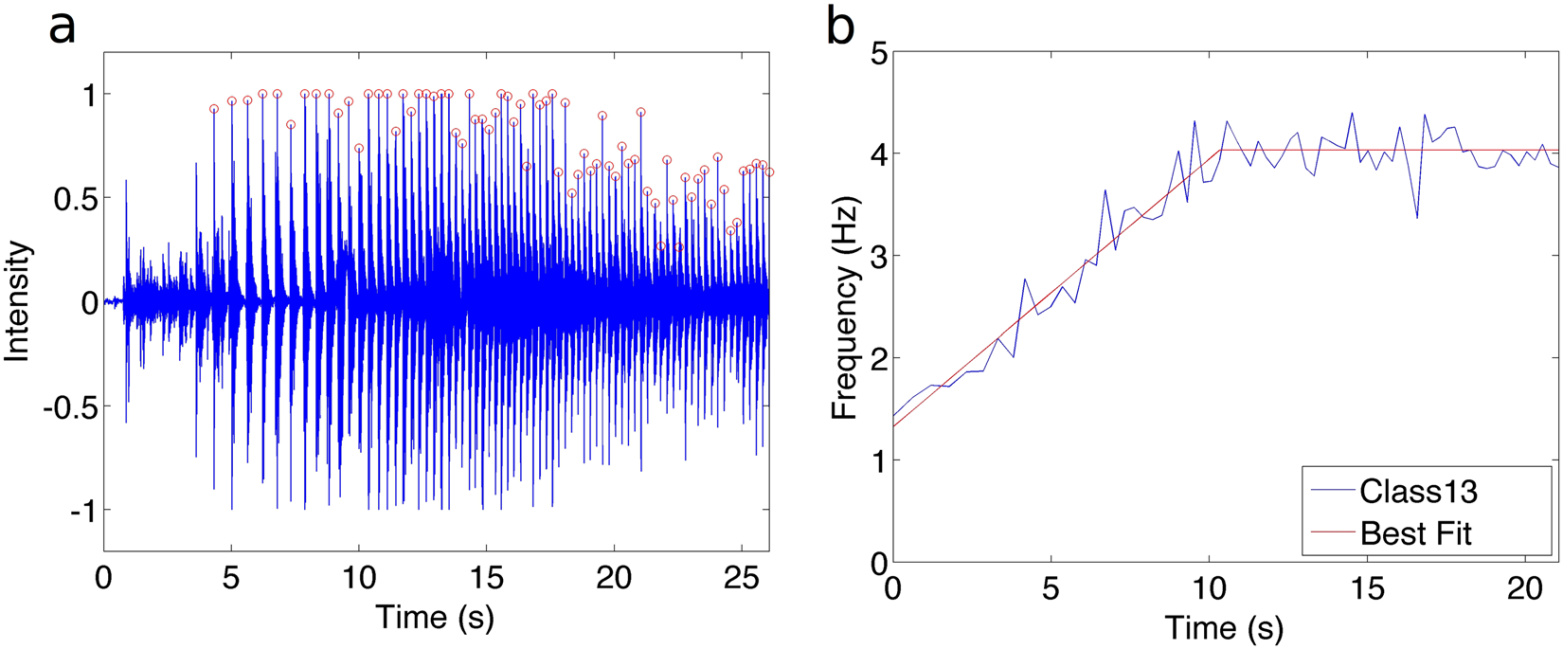
(**a**) Example for a typical trial of the group clapping experiment. Audio intensity (blue) peaks at the time of the group clap (circles) and are extracted using a purpose built peak-finding algorithm, customized for the audio features of each experiment. (**b**) Frequency versus time for a typical trial of the group clapping experiment. Frequency of clapping (blue) increases in time, then plateaus. Piecewise regression (line of best-fit, then a constant line) is shown in red. Slope of increasing portion is 0.262 Hz/s.

For all group recordings analyzed, synchronous clapping was established very quickly, typically after only a few seconds. Once collective synchrony was established, in all 32 experiments the collective clapping frequency increased in time (to varying degree), followed typically by stabilization at some higher frequency. The plateau effect is likely a result of individual biomechanical limitation at high clap frequency (as in tapping experiments^15,19^). A qualitatively high level of synchrony was maintained throughout experiments, except at high frequencies impacted by biomechanical limitation. In order to focus on the increase in frequency specifically, a piecewise linear regression was performed on group frequency for each trial, composed of a time interval of increase, a breakpoint, and then a time interval of constant frequency (such that the fit is continuous across the breakpoint). For each trial, the regression was performed by testing the breakpoint at each clap interval, calculating a line of best fit before the breakpoint, then imposing the same frequency value beyond the breakpoint. The optimal breakpoint was then determined by minimizing the root mean square error to the data. Figure 1b shows the evolution of frequency after synchrony is established, together with the piecewise regression best fit, for one group trial (40 participants). Using this approach, the subset of each trial showing frequency drift (versus plateau) was isolated for analysis. On average, 42.1 claps per trial were retained for analysis per trial. All results of regression are contained in Table S1 in the supplementary information. For 31 of the 32 retained trials, the frequency increase was statistically significant (*p* < 0.0026 in all 31 cases). In the remaining case, there was a small increase in frequency (slope = 0.004 Hz/s), but this was not statistically significant (*p* = 0.38). Emerging from the collective synchronization process, average initial frequency across all (unentrained) group trials was 1.9 Hz, close to the optimal human entrainment rhythm of about 2 Hz (120 bpm)^20^. Average coefficient of determination across trials was *R* ^2^ = 0.784. The frequency increase was better described by a linear model (versus nonlinear). Frequency time series for all trials is shown in Fig. S1.

**Figure 2:**
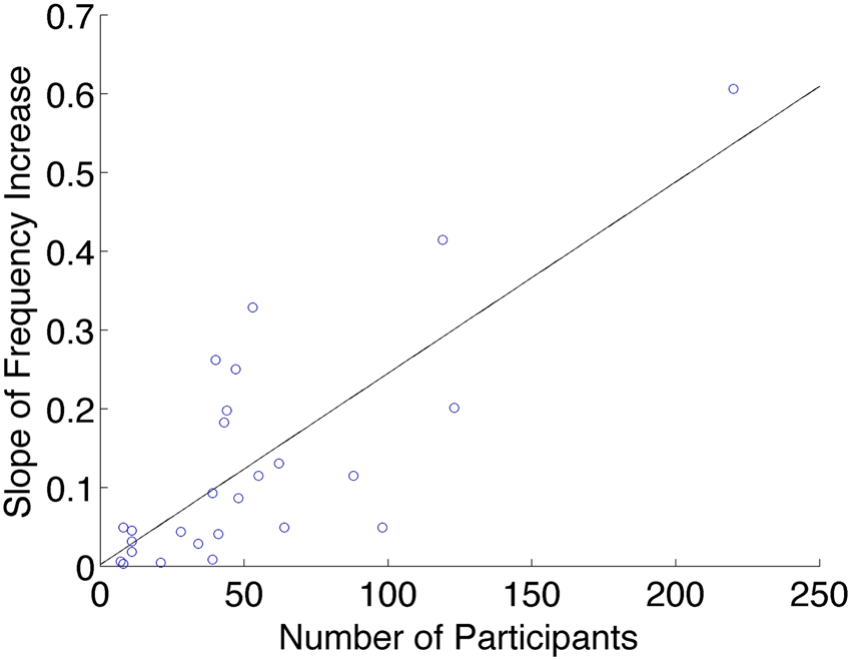
A scatterplot of the slope of frequency increase (Hz/s) versus group size for the 32 trials, with least squares regression line.

Larger group size was associated with a more rapid increase in frequency. Figure 2 shows a scatterplot of the slope of frequency increase for the 32 trials as a function of group size *N*, showing significant positive correlation (*R* ^2^ = 0.78, *p* = 2.45 × 10^−6^).

To investigate the effect of musical training, one trial was conducted for a different population: upper-year students in an Advanced Rhythm class from the music department at St. Francis Xavier University. This group showed no statistically significant increase in frequency (see Fig. S2); the small positive slope (0.0135 Hz/s) occurred only for a short period of time (the breakpoint occurs less than 10 seconds into the trial), and was not statistically significant (*p* = 0.14). Thus, it appears that musical training could mitigate to a large extent the tendency to increase the collective clap frequency. However, whether this was due to different rhythmic characteristics of the population (e.g., distribution of timing errors), or to a greater awareness of timekeeping in general (leading to a collective tendency to correct for frequency drift), is not clear.

To explore the mechanisms of inter-individual interaction taking place during collective rhythmic clapping, three main observations are retained from the experiments that are to be replicated in a valid model. First, frequency increases after synchrony is attained for all group sizes. Second, the increase in frequency is approximately linear. Third, the clapping frequency increases more rapidly as group size increases. We examine two distinct hypotheses to explain the observed frequency increase.

### Hypothesis I

One possible explanation for the observed collective frequency increase is that, on average, individuals tend to speed up when trying to maintain a fixed rhythm. Then, the collective frequency would simply reflect the averaging of individual rhythmic tendencies. A similar phenomenon has been observed in finger-tapping studies of sensorimotor synchronization, termed *negative mean asynchrony* (NMA). Here, individuals synchronizing with a metronome tend to tap a few tens of milliseconds prior to the tone onset^15^, yet are typically unaware they are doing so. The NMA has been found in most metronome-based tapping studies, although the underlying cause is not completely understood (but see ref.^21^ for various hypotheses). There tend to be large individual variations in the magnitude of the NMA^22^, and musical training reduces or eliminates it^23^, as do experiment modifications such as explicit auditory feedback and rhythmic subdivision (either via tones or movements) of the timing interval^22,24^. Because of the various factors upon which the existence of the NMA depends, it is not obvious that the applicability extends to clapping (which involves, among other differences, other body movements to subdivide the timing interval), but it does provide the simplest explanation of the observed frequency increase. As a first investigation of this hypothesis, we tested rhythmic clapping of 5 individuals entrained to a metronome, to see if they would increase frequency without any interaction effects. At three different entrainment frequencies (70, 90, and 110 bpm) and for one minute of measurement, average change in frequency per interval (averaged across time and individuals) was negative (see Table S2). This indicates a slowdown in clapping frequency in these trials, rather than increase, although the tendency is very slight (−0.00041, −0.00089, and −0.00071 Hz (average, per interval) for 70, 90, and 110 bpm respectively). Pooling the 5 participants, the distribution of changes in frequency (i.e., timing errors) was approximately normally distributed, and standard deviations ranged from 0.070 to 0.097 Hz across the 3 entrainment frequencies. This experiment does not provide compelling evidence for Hypothesis I, but individuals entrained to a metronome clapping in isolation is a slightly different task than clapping in unison with other individuals; individuals can globally adjust drifting frequency in a way that is not as straightforward in groups.

To formalize and test this hypothesis, a mathematical model (model I) was constructed. Consider *N* oscillators indexed by *i*, each with phase *x*_*i*_ ∈ [0,1] and frequency *ω*_*i*_, such that (*dx*_*i*_)/(*dt*) = *ω*_*i*_. If *x*_*i*_ ‘fires’ (i.e., reaches 1, indicating a clap), it resets to 0, and frequency is then discretely updated via

1 $${\omega }_{i}(t+{\rm{\Delta }}t)={\xi }_{i}+\frac{1}{{T}_{avg}+{t}_{avg,last}+{T}_{avg}-t}\mathrm{.}$$

Here, *ξ*_*i*_ is a Gaussian error term with mean *μ* and standard deviation *σ*; assigning a negative mean represents the presence of an NMA tendency. Further, ${T}_{avg}=\frac{1}{N}{\sum }_{j=1}^{N}\frac{1}{{\omega }_{j}(t)}$ is the average group period, *t*_*avg*,*last*_ is the average last firing time for the *N* oscillators, and *t* is time (from the start of the simulation). Thus, the *t*_*avg*,*last*_ + *T*_*avg*_ − *t* terms represent an adjustment of firing time to match the anticipated average firing time of the collective. For example, if an individual fired early relative to the group average, *t*_*avg*,*last*_ + *T*_*avg*_ − *t* > 0, and then *ω*_*i*_(*t* + Δ*t*) is reduced accordingly. For a given model experiment, the set of claps for each individual *i* form a discrete sequence of periods, written as ${T}_{i}^{k}$, where *k* indexes through the sequence of claps for individual *i*.

**Figure 3:**
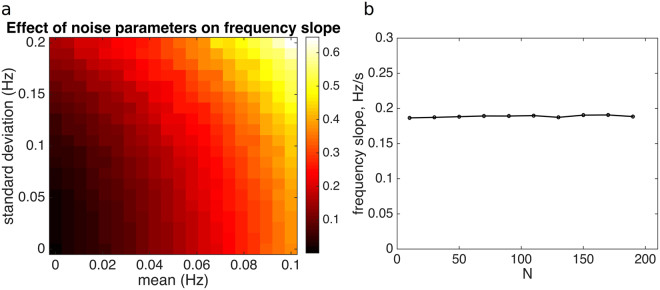
Simulation results for Model I. (**a**) Slope of frequency increase for mean and standard deviation of Gaussian error *ξ*_*i*_ for simulations with *N* = 30. (**b**) Slope of frequency increase is invariant to group size, *N*. Simulations were run with *μ* = 0.05 and *σ* = 0.1.

To investigate the effect of error parameters on the frequency slope, simulations of model I were performed. Groups were initialized in perfect synchrony and oscillating at 2 Hz, then evolved for 10 seconds (Δ*t* = 0.0025 sec), with output averaged over 10 repetitions for each parameter combination. To initialize period data, oscillators are artificially imposed to oscillate at initial frequency 2 Hz for two cycles. In addition to the collective frequency, *group synchrony* was also calculated via $q(t)=|\frac{1}{N}{\sum }_{j\mathrm{=1}}^{N}{e}^{2\pi i{x}_{j}}|$, such that *q* = 1 corresponds to perfectly synchronized groups (all clapping in unison), and *q* = 0 to completely asynchronous groups. Parameter ranges of *μ* ∈ [0,0.1] (with the upper limit of 0.1 corresponding to an NMA of about 25 ms at 2 Hz) and *σ *∈ [0,0.2] all permitted a high degree of synchrony (*q* > 0.9 for all combinations) but extending beyond this range led to a relatively sharp reduction in synchrony. Because the experiments were characterized by a highly synchronous behaviour, parameter values beyond those in the chosen range are incompatible with the experimental observations. In Fig. 3a, frequency slope is plotted across the combinations of *μ* and *σ*. The resulting range of frequency slopes contains those from the experimental data. Increasing the magnitude of *μ* (i.e., the size of the NMA) leads to steeper frequency increases, as expected. Additionally, increasing the variability in timing error through the range of *σ* also led to a slightly steeper frequency increase.

Model I is therefore able to recover realistic frequency increases in simulations. However, frequency increases are concave up, with this nonlinearity becoming more pronounced as the overall frequency slope increases. Also, varying group size *N* has no effect on the frequency slope (Fig. 3b), in contrast to the experimental observations. Thus, Hypothesis I is not compatible with two main observations carried forward from the experiments.

### Hypothesis II

A second explanation of the observed frequency increase is that it arises out of the mode of interaction among individuals (as opposed to simply reflecting the individual tendency to increase frequency, as in Hypothesis I). If the frequency increase were a product of interaction, then it should be present for pair experiments, but absent in single-person experiments. Indeed, as discussed in the previous section, no evidence was found for the frequency increase for individuals. Adding a second person, however, did lead to observable frequency increases. We begin with these *N* = 2 experiments to deduce the interaction mechanism, employing a bottom-up modeling approach.

#### Results for pair experiments

For the three pair groups under 4 entrainment conditions, clap frequency increased for both individuals in every trial (12 of 12 instances each, 24 in total). Piecewise linear regression (as in the large group recordings) gave a positive slope for all 24 individual trials, most of which were statistically significant (*p* < 0.01 for 18 trials, *p* ∈ [0.08,0.09] for 4 trials, *p* > 0.1 for 4 trials). Slope values ranged from 0.0016 to 0.0101 Hz/sec, with an average increase of 0.005 Hz/sec (about a 0.3 Hz increase over the minute duration of the experiment).

To specify the interaction within pairs, we investigated how individuals adjust their clap timing in response to differences in timing with the partner at the previous clap instance. That is, for individual *i* (*i* = 1,2) how the period difference ${\rm{\Delta }}{T}_{i}={T}_{i}^{k+1}-{T}_{i}^{k}$ depends on clap timing *t*_*j*_ − *t*_*i*_ (*i*,*j* = 1,2, *i* ≠ *j*) relative to the partner, measured at each clap instance. As such, negative values of *t*_*j*_ − *t*_*i*_ correspond to a focal individual *i* who is late relative to the partner, thus receiving the stimulus directly before clapping (and positive values to an early individual, receiving the stimulus just after clapping). Similarly, positive values of Δ*T*_*i*_ correspond to individuals increasing their clap period following stimulus (and negative values to those decreasing their clap period).

**Figure 4:**
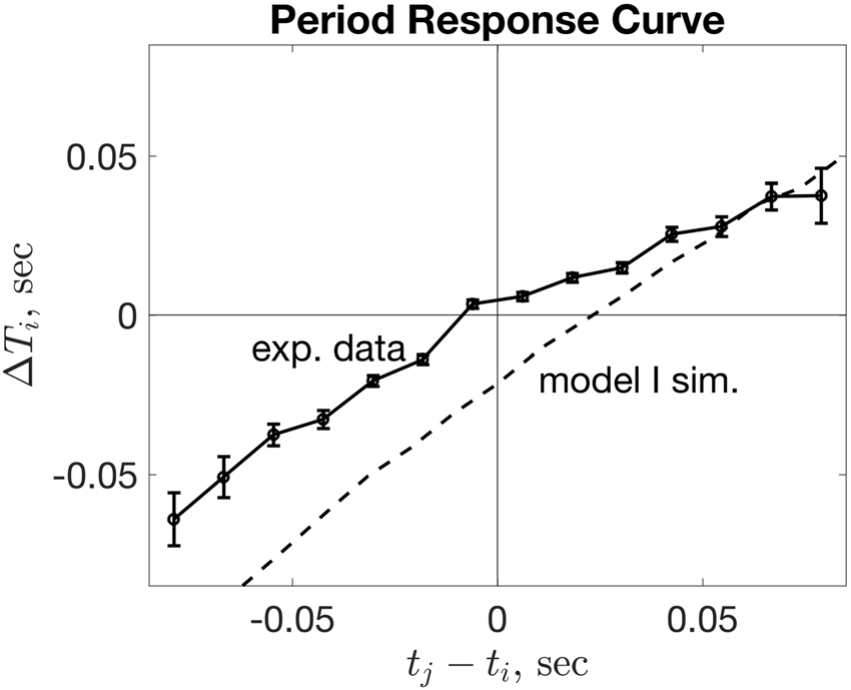
The period difference ${\rm{\Delta }}{T}_{i}={T}_{i}^{k+1}-{T}_{i}^{k}$ as a function of clap timing *t*_*j*_ − *t*_*i*_ at the current clap instance, measuring the response of each individual in clap timing at the next step caused by differences in timing with the partner at the current clap instance. The solid line corresponds to experimental data (with standard error bars), and the dotted line to simulations of Model I.

Pooling all pair experimental data, the resulting plot (Fig. 4) gives a period-based analogue to a phase response curve for the auditory stimulus provided by the partner clapping. The response curve shows an asymmetric response in period adjustment depending on whether the stimulus is received directly before, or after, an individual claps: receiving the partner auditory stimulus just after an individual claps results in a weaker adjustment than receiving the stimulus just before. If individuals compensated perfectly for the discrepancy in timing at the next interval, the period response curve would be linear with slope 1. Fitting a linear regression to the negative and positive portions of the *t*_*j*_ − *t*_*i*_ axis gives slope values of 0.90 and 0.53 respectively, and so individuals in these experiments compensated almost twice as strongly based on an early stimulus than a late stimulus, and compensation was proportional to the magnitude of timing difference. These observations inform the specification of the inter-individual interaction contained in Model II.

The observation that individuals who are nearing their planned clap time are more sensitive to the claps of others, versus having just completed a clap, is mirrored in a set of tapping experiments^25^. In these experiments, participants attempting to match a sequence of metronome tones were subject to distractor tones. The attraction (or phase shift) to the distractor was stronger when the distractor tones preceded the target tone, than when they lagged behind the target tone.

We note that under the assumptions of Model I with *N* = 2, the reduction in period (increase in frequency) caused by the NMA is uniform across timing differences with neighbours, and so the period response curve (as in Fig. 4) takes the form of a straight line with slope 1, shifted downward by an amount proportional to the size of the NMA. An example response curve from simulations of Model I with *N *= 2, *μ *= 0.1, and *σ* = 0.1, (averaged over 5000 simulations of 5 seconds) is shown in the dotted line in Fig. 4. This discrepancy from the observed period response in experiments (solid line) provides further evidence that Hypothesis I is unsuitable.

#### Model II

Model II uses the same basic framework as that of Model I, but with the modification that after a clap, individuals simply attempt to copy their own previous period, with some zero-mean error (i.e., no NMA) with standard deviation *σ*_*i*_. Then 1 becomes

2 $${\omega }_{i}(t+{\rm{\Delta }}t)={\xi }_{i}+\frac{1}{t-{t}_{i,last}},$$

where Gaussian noise *ξ*_*i*_ has zero mean, and standard deviation *σ*_*i*_, *t* is the current time of firing, and *t*_*i*,*last*_ is the time of last firing. A modification of a commonly used excitatory coupling^2,26^ is used to model interaction effects. When *x*_*i*_ fires, then for all *x*_*j*_, *j* ≠ *i*, *x*_*j*_ = min (1,*x*_*j*_ + *f*(*x*_*j*_)(*ε*)/(*N*)), where *f*(*x*_*j*_) is piecewise constant function such that *f*(*x*_*j*_) > 0 for *x*_*j*_ ∈ [0.5,1) and *f*(*x*_*j*_) < 0 for *x*_*j*_ ∈ ( 0,0.5). Thus, ‘early’ individuals who are in the first half of their period undergo a reduction in phase (slowing down), and ‘late’ individuals undergo an increase in phase (speeding up). The ratio between the two constant values is determined by the slope ratio (0.53/0.90) in the period reduction curve for the pair experiments, and accordingly,

3 $$f({x}_{i})=\{\begin{array}{ll}1 & \,\,{x}_{i}\ge 0.5\\ -0.59 & \,\,{x}_{i} < 0.5\end{array}$$

Thus, once an individual fires (claps), all other individuals interpret that as a signal to modify phase, and since individuals attempt to copy thier own previous period, indirectly modify period and frequency. The magnitude of the phase shift engendered by the clap of another individual is controlled by the parameter *ε*, and is scaled by the population size *N*. Together with *σ*, these three parameters specify the model.

**Figure 5:**
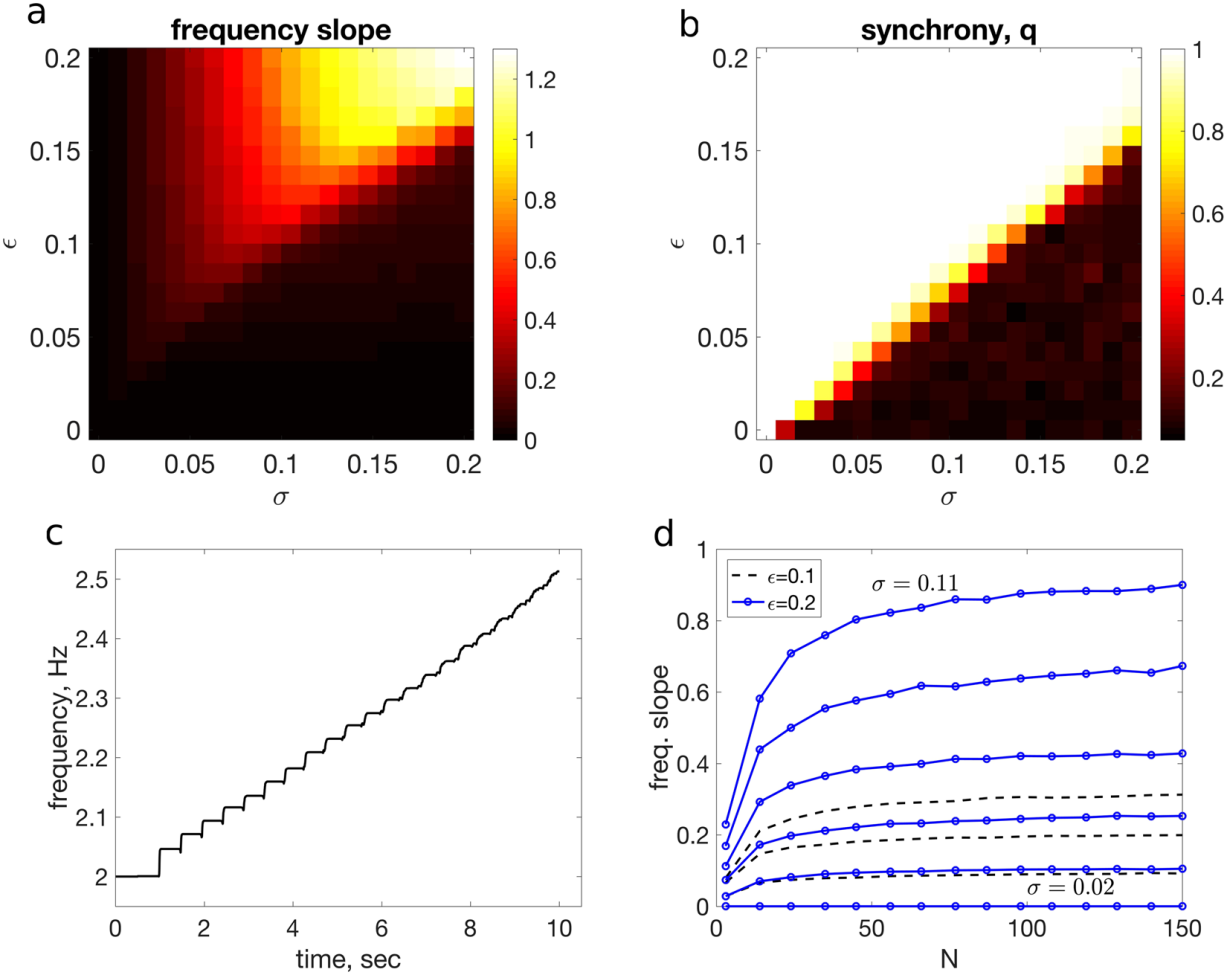
Simulation results for Model II. (**a**) Slope of frequency increase in simulations of Model II with *N* = 100, averaged over 10 trials, for parameter combinations of *ε* ∈ [0,0.2], and *σ* ∈ [0,0.2] (sec). (**b**) Synchrony values *q* for Model II with *N* = 100, averaged over the 10 second trial duration, and over 10 trials. (**c**) Example of approximately linear trend of frequency versus time for simulations of Model II (averaged over 10 simulations) with parameters *ε* = 0.05, *σ* = 0.05 and with *N* = 100. (**d**) Example of slope of frequency increase as a function of group size *N*. Values shown for *ε* = 0.1 (dashed) and *ε* = 0.2 (circles) through a range of *σ* values from 0 to 0.11.

Simulations of Model II were performed using the same initial conditions, timestep and trial length as in Model I simulations. In contrast to Model I, the frequency evolution is dependent on group size *N*, and so a three-dimensional parameter search (across *N*,*ε*, and *σ*) was undertaken to assess model behaviour. Across parameter ranges, the frequency increase (caused by the asymmetric phase shift in *f*(*x*_*i*_)) is observed. Increasing the variation in timing error *σ* causes a faster increase in frequency (higher ‘frequency slope’). Similarly, but to a lesser extent, increasing the coupling parameter *ε* also increases frequency slope. In Fig. 5a, the slope of the regression fit to frequency (as in Fig. 3a) is shown over a range of *ε*,*σ* pairs, with *N* = 100. The corresponding synchrony values *q* are shown in Fig. 5b, where strong synchrony is established provided the coupling parameter *ε* is sufficiently large relative to the timing variation *σ*; otherwise, groups are asynchronous. For the case of *N* = 100 in Fig. 5b, the synchrony threshold occurs approximately when *ε* > 0.75*σ*. The same qualitative structure in Fig. 5a and b persist across the range of *N* values. Extending beyond the range of *ε* shown has little to no effect on slope or synchrony values, as the phase interactions saturate. For a given set of (*ε*,*σ*) parameters, the trend of frequency versus time is approximately linear, consistent with experimental observations. An example (averaged over 10 simulations) for *ε* = 0.05, *σ* = 0.05 and *N* = 100 is shown in Fig. 5c.

Simulations of Model II further reveal that, for a given pair of parameters *ε*,*σ*, frequency slope increases as group size *N* increases. That is, larger groups more rapidly speed up their clapping frequency. This is not a result of more individuals leading to a larger accumulation of phase shifts and thus a sharper increase in frequency, as phase interactions are normalized by *N*. Nevertheless, in Fig. 5d, with *ε* fixed at two different values and ranging through *σ* ∈ [0,0.11], frequency slope monotonically increases in each case with *N* (with the effect saturating with larger *N*). Although the shape of this curve persists across parameters, the specific relationship is governed by the *ε*,*σ* pairs: increasing *ε* and *σ* both lead to a more pronounced frequency slope increase as *N* increases.

The faster frequency increase induced by larger group size *N* is actually due to the larger range of phase errors occurring with larger groups. This larger range leads to a faster time to first ‘fire’, setting off a chain reaction of successive firings and a faster overall frequency increase. Because of the asymmetric coupling in *f*(*x*_*i*_), this larger range effect is not completely counteracted by the corresponding slow-down effect of late firings on other individuals, and so the overall frequency increase persists.

## Discussion

Humans clapping in unison provides a tangible, simple example of self-organized collective behaviour. Nevertheless, nontrivial dynamics arise after synchronous behaviour is established. The mechanisms of interaction, determined stepwise from the individual to the collective, uncover a behavioral asymmetry in human aural interactions that explain the group-level observations of frequency increase, and how this increase scales with group size. Beyond the insights about human synchronous behaviour, this model system carries instructive value for ease in experimental replication, and mathematical tractability.

We found that humans under instruction to clap in unison will also, reliably, increase the frequency of clapping. This increase occurs more quickly as the size of the group increases. To explain this collective tendency to speed up, two competing hypotheses were presented. In the first, mirroring observations of finger-tapping studies, it was hypothesized that individuals, on average, have a tendency to speed up (i.e., ‘rush the beat’), and so, under the most simple framework for synchronization (matching the average behaviour of the group), the collective tendency to increase frequency is a reflection of the sum of individual behaviours. However, we found that although this hypothesis could lead to realistic frequency increases given the variability in timing observed at the individual level (and estimations of the hypothesized negative mean asynchrony from tapping studies), a number of inconsistencies arose. First, single person clapping experiments showed no such tendency to increase frequency in time (and in fact individuals on average had a slight tendency to slow down). Second, our simple averaging model showed that frequency increases were nonlinear, whereas experimental observations showed a linear trend to this increase. Further, the model showed no dependence of the rate of frequency increase on group size, as seen in experiments. Finally, the period response curve for simulated pairs did not match the qualitative structure observed in pair clapping experiments.

Instead, we alternatively hypothesized that the aural interactions among individuals lead to the frequency increase. Here, the pair-based observation of asymmetric response to aural information (significantly weaker for information following, rather than preceding, one’s own clap) was used to specify a phase-modifying interaction among individuals, parametrized from the data. A conceptual coupled-oscillator model was then constructed based on this interaction, completely specified by group size, and two parameters governing interaction strength and individual variability. This model recovered the approximately linear increase in frequency, and the tendency for the frequency increase to occur more quickly for larger groups. Furthermore, results are consistent with the single-person clapping experiments: absent the frequency-increasing interactions, no increase is expected, nor observed.

Based on this analysis, it is clear that the tendency for humans clapping in unison to speed up is an epiphenomenon of inter-individual behaviour, and not representative of some collective desire to increase, e.g., noise intensity. Certainly, our experiments (pairs, and larger groups) took place in an environment where there was no clear explanation for a collective desire to increase noise (as possibly in an audience context). The frequency increase is noise-induced, arising due to the imperfect timing capabilities of individuals, and the subsequent small adjustments to timing made to maintain unison with others. With a larger group, one expects a larger range of timing errors, and those early individuals initiate a phase advance in others that sequentially leads to the faster increase in frequency. With the variability in timing frequency observed in the individual experiments (standard deviation *a* = 0.07 to 0.09 Hz), simulated frequency increases were consistent with observed values. Reducing the timing variability of group members would then reduce the tendency for frequency increase, and stands as one explanation for why the musically trained group did not show a statistically significant tendency to speed up.

In the interaction model (Model II) presented here, strength of interactions was normalized by the size of the group, eliminating the effect of larger groups leading to steeper frequency increases trivially through a larger sum of phase jumps at fixed coupling strength. Indeed, the precise way in which the sensitivity to aural information from others scales with group size is not known. Determining this relationship would refine the dependence of frequency slope on group size.

The error-induced frequency increase offers further explanation for the phenomenon of transient synchronized clapping in audiences of certain cultures^12^. When synchronous clapping is established, the timing errors lead to a steady increase in clapping frequency, until individuals cannot physically maintain the rhythm and synchrony is lost, and the process repeats. Indeed, the authors noted that the clapping period slowly decreased (and thus frequency increased) as synchronization was lost.

Beyond the specific instance of humans clapping, these results have application in understanding musical rhythmic behaviour, and how information spreads in collectively interacting human groups. More broadly, determining the inter-individual interactions that explain group level observations is of primary importance to understanding complex systems. The framework of systematic modeling built upon sequential experimental data from the individual to collective will be important in unraveling this complexity.

## Methods

Recordings were made of individuals, pairs, and groups, drawn from the student body of St. Francis Xavier University, and all participants voluntarily took part in the experiment. Classes of students were used for group recordings, and so group size in each case was determined by class attendance, ranging from 7 to 220 individuals. Individuals who had participated in the experiment in a previous class were dismissed. The experiments described in this manuscript were approved by the Research Ethics Board of St. Francis Xavier University, and all experiments were performed in accordance with the relevant guidelines and regulations. Informed consent was obtained from all participants. Audio data was collected using a Tascam DR-100 portable digital audio recorder, and then processed using Audacity. Analysis of audio data to extract time series of collective claps (appearing as peaks in the waveform) was performed in MATLAB using purpose-built software. After extraction of claps, manual audio verification was performed against original recordings to ensure correctness. An example waveform with extraction of peaks is shown in Fig. 1a.

### Group recordings

33 university classes of varying size were selected for experiments, on the basis of reducing likelihood of overlap of students. At the start, individuals who had previously taken part in the experiment (or who refused participation) were asked to leave. Those remaining were instructed to clap together in synchrony following the signal of the researcher (blowing a whistle). Lights were turned off for the duration of the experiment to reduce or eliminate visual interaction among participants. No initial entrainment via metronome was given for the group experiments. One group trial was excluded from analysis due to a lack of adherence to the instructions given.

### Single-individual and pair recordings

To examine characteristics of individuals clapping without interaction, single individuals were prompted to clap in time with a metronome, set at 70, 90, and 110 beats per minute (bpm). In each case, after an initial period of entrainment, the metronome was turned off and individuals were recorded clapping for approximately one minute. In another set of experiments, individuals were asked to clap in rhythm with no initial entrainment to a metronome. For pair recordings, two individuals were brought into a room, facing back to back to eliminate visual cues. Pairs were asked to clap together rhythmically, and were entrained to the metronome as in the single-participant case, and then also with no entrainment. Pairs were recorded for approximately one minute after entrainment, using a separate device for each individual. Three pair groups (6 individuals) were recorded in total, four times each with initial entrainment at 70, 90, and 110 bpm, and without entrainment.

## Electronic supplementary material

Supplementary Information
