# Supplementary material for: Groups clapping in unison undergo size-dependent error-induced frequency increase
Source: Sci Rep. 2018 Jan 16;8:808. doi: 10.1038/s41598-017-18539-9 (PMC5770382; doi:10.1038/s41598-017-18539-9)
Supplement: Supplementary file 1 — Supplementary Information [file 41598_2017_18539_MOESM1_ESM.pdf]

Groups clapping in unison undergo size-dependent error-induced frequency increase

M. Thomson, K. Murphy and R. Lukeman.

Supplementary Information

Table 1: Frequency statistics, all trials

Group	N	Slope (Hz/s)	Intercept (Hz)	R^2	p-value	Max. Freq. (Hz)	Claps
1	50	0.049999	1.7855	0.75174	2.2847×10^{-5}	2.441	24
2	50	0.11974	2.2129	0.95181	1.1692×10^{-22}	3.771	43
3	52	0.0092506	3.2260	0.40308	3.1537×10^{-7}	3.700	150
4	26	0.014654	2.3087	0.53851	1.0606×10^{-7}	2.789	85
5	59	0.10060	2.1049	0.80521	3.1955×10^{-9}	3.510	36
6	98	0.049097	1.7021	0.90280	2.3317×10^{-27}	3.195	72
7	8	0.0033020	1.0593	0.73515	1.7990×10^{-9}	1.219	49
8	43	0.18241	2.1995	0.95358	3.2063×10^{-9}	3.338	17
9	62	0.13065	1.9358	0.96348	1.8699×10^{-20}	3.394	35
10	28	0.043877	3.3157	0.82467	2.0994×10^{-8}	3.626	30
11	34	0.028605	2.2027	0.89138	8.2244×10^{-19}	2.715	52
12	7	0.0061918	1.7475	0.37974	0.0018092	2.089	65
13	40	0.26202	1.3255	0.96292	6.8427×10^{-17}	3.937	29
14	8	0.049413	1.4232	0.91645	8.2564×10^{-10}	2.095	23
15	21	0.0048193	1.7006	0.53567	7.5420×10^{-8}	2.008	88
16	11	0.018297	2.1212	0.44202	0.00036185	2.683	61
17	41	0.040998	2.6238	0.54211	0.00017345	3.527	43
18	44	0.19780	1.8174	0.97132	2.8575×10^{-20}	3.774	32
19	48	0.086481	1.7832	0.84712	2.2082×10^{-13}	3.309	45
20	53	0.32884	1.3509	0.97811	1.7210×10^{-16}	4.196	24
21	123	0.20121	2.0583	0.94572	3.2656×10^{-13}	3.843	26
22	11	0.045406	1.7760	0.88946	4.1498×10^{-13}	2.521	36
23	88	0.11490	2.3158	0.74817	8.5311×10^{-7}	4.249	32
24	220	0.60628	1.7146	0.96289	3.4807×10^{-8}	4.132	14
25	119	0.41453	1.1883	0.94368	1.4822×10^{-11}	4.628	23
26	47	0.25040	1.3744	0.96676	2.5365×10^{-16}	4.081	27
27	39	0.092793	1.5284	0.96673	4.6691×10^{-33}	3.495	55
28	11	0.031805	1.7438	0.89226	6.1641×10^{-13}	2.247	35
30	64	0.049307	2.0562	0.62486	0.0024578	2.290	21
31	39	0.0084791	2.1523	0.16941	0.37965	2.288	29
32	55	0.11482	1.2384	0.92419	1.6948×10^{-25}	3.947	59
Average:	51.6	0.11797	1.9062	0.78487	0.012403	3.195	43.9
Std. Dev.	42.4	0.13470	0.51568	0.21626	0.067052	0.819	26.9

Table 2: Single-participant experiment statistics

Trial	Mean of ΔF (Hz)			Std. Dev. of ΔF (Hz)		
	70 BPM	90 BPM	110 BPM	70 BPM	90 BPM	110 BPM
1	−0.0011818	−0.0010109	−0.00050427	0.052556	0.10136	0.080022
2	−0.00028748	−0.0015433	−0.0015514	0.052262	0.086990	0.10697
3	−0.0010556	−0.00014763	−0.00076833	0.052528	0.076211	0.091323
4	−0.00037591	0.00073245	−0.0017837	0.066925	0.071863	0.093979
5	0.00083046	−0.0024765	0.0010353	0.10970	0.095346	0.10962
Average	−0.000414	−0.00088	−0.00071	0.0704	0.0869	0.0971

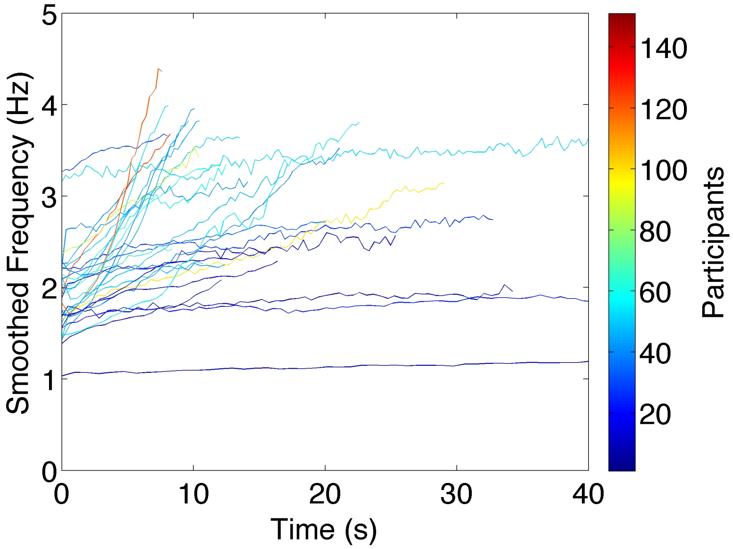

Figure 1: Frequency of clapping for all trials, for the portion of increasing frequency retained for analysis. Color indicates the number of participants for each experimental trial. For this visualization, frequency data were smoothed using a 3-point moving average.

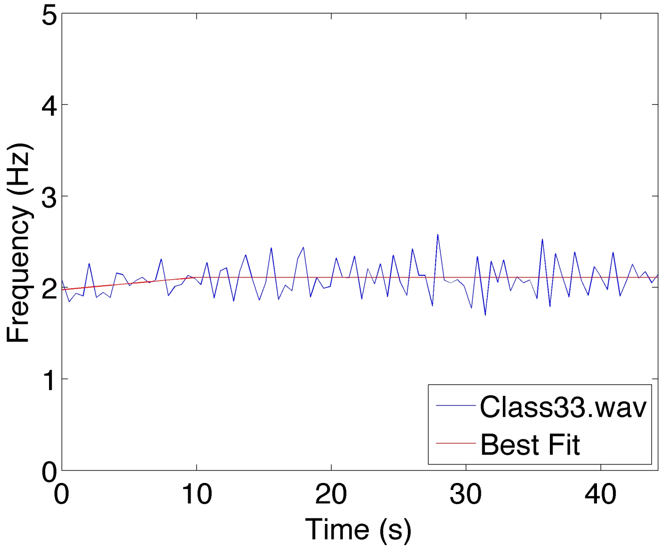

Figure 2: Frequency of clapping (blue) from trial on musically trained individuals, together with piecewise regression (red). No statistically significant increase in frequency is observed.
